# Editorial: Nutrition and oral biology in health and disease

**DOI:** 10.3389/fnut.2023.1178502

**Published:** 2023-03-27

**Authors:** Fernando Capela e Silva, Gemma Bridge, Elsa Lamy, Paula Midori Castelo

**Affiliations:** ^1^Department of Medical and Health Sciences, School of Health and Human Development, MED-Mediterranean Institute for Agriculture, Environment and Development & CHANGE-Global Change and Sustainability Institute, Pólo da Mitra, University of Évora, Évora, Portugal; ^2^Business School, York St John University, York, United Kingdom; ^3^MED-Mediterranean Institute for Agriculture, Environment and Development & CHANGE-Global Change and Sustainability Institute, Pólo da Mitra, University of Évora, Évora, Portugal; ^4^Department of Pharmaceutical Sciences, Universidade Federal de São Paulo (UNIFESP), Diadema, Brazil

**Keywords:** food choices, diet, nutrition, oral biology, oral health

Daily food choices have an impact on global human health as good nutrition allows the body to function properly. People with healthy eating patterns are less likely to develop serious illnesses such as heart disease, type 2 diabetes, obesity, and some types of cancer, which can translate into a longer life expectancy. Healthy lifestyles, including balanced and nutritionally adequate diets, can also help prevent diseases of the oral cavity, such as tooth decay, gingivitis, periodontal disease, and oral cancer. Recent research on the relationship between diet and dental caries emphasize the importance of limiting free sugars in the diet, including sucrose, glucose, and lactose, for the prevention of tooth decay; in addition, studies have pointed out the importance of soft drinks as the most important dietary factor related to enamel erosion. In this sense, dietary guidelines suggest the adoption of a diet low in fats and free sugars, but rich in fruit, vegetables, and fibers as a protective measure against the development of oral diseases. Proteins and vitamins (both fat- and water-soluble), as well as calcium, phosphorus, and fluoride, are essential nutrients for the development, maturation, and protections of oral tissues at all stages of life.

It is worth mentioning that this is a two-way relationship: oral health can also affect nutrition. Healthy oral tissues—teeth, bones, mucosa, muscles and joints—are vital for proper chewing of food and ensure that ingested food is broken down into small pieces and prepared for digestion. The first steps of digestion occur in the mouth, with the humidification of the bolus by the saliva, digestion of starch, and its buffering effect. Taste receptors have a direct influence on sensory capacities, with an impact on food choice and acceptance and, consequently, on nutrition. Saliva also plays a role in maintaining oral health helping to keep the integrity of soft and hard tissues, mastication, taste, and texture perception, swallowing and initial digestion. On the contrary, oral problems influence nutrition by limiting food choices: tooth decay and tooth loss can lead to limited masticatory function and reduced food intake, increasing the risk of chronic diseases, malnutrition, and low wellbeing and quality of life, especially in the elderly population.

The World Health Organization (WHO) considers that having good oral health is essential for having good quality of life (1) and defines malnutrition as “*deficiencies, excesses or imbalances in a person's intake of energy and/or nutrients. The term malnutrition covers two broad groups of conditions. One is ‘undernutrition'—which includes stunting (low height for age), wasting (low weight for height), underweight (low weight for age), and micronutrient deficiencies or insufficiencies (a lack of important vitamins and minerals). The other is overweight, obesity and diet-related non-communicable diseases (such as heart disease, stroke, diabetes, and cancer)*” (2). It should also be noted that socioeconomic inequalities and food insecurity are other factors that profoundly affect lifestyle, food choices, and general and oral health. Knowledge of oral biology within the scope of food and nutrition sciences is important, prioritizing interdisciplinary approaches to ensure that individuals maintain adequate nutrition and a good state of oral health.

Gingivitis is an inflammatory condition that involves the gums and results from the biofilm accumulation. If left untreated, it can progress to periodontitis and a range of negative health outcomes including, loss of alveolar bone that supports the teeth, increased probing depth, tooth loss, chewing difficulty, speech difficulties, halitosis, and self-esteem issues. The main risk factors for periodontitis are diabetes and smoking, but a poor diet and inflammatory conditions may also increase the risk of periodontal disease. Jiaxin et al. evaluated the relationship between moderate/severe periodontitis and plasma levels of elaidic acid, a major trans-fatty acid component, in American adults, hypothesizing that an inflammatory dietary pattern is linked to the prevalence of periodontitis. The authors used the National Health and Nutrition Examination Survey (NHANES; years 2009–2010) to screen a total of 1,610 people. The results of multiple logistic regression analysis showed a significant association between plasma elaidic acid level and moderate/severe periodontitis, providing further support for the recommendation to limit trans-fatty acid consumption.

Considering that two-thirds of the US population suffers from magnesium deficiency, Li, Wen et al. investigated the link between dietary magnesium intake and periodontitis. The authors collected data from the National Health and Nutrition Examination Survey (NHANES) database from 2013 to 2014 and observed that dietary magnesium intake was negatively associated with the incidence of periodontitis; thus, the increase in magnesium intake through dietary modification may help reduce the prevalence of periodontitis.

Also in the field of periodontitis, the purpose of Li, Liu et al. study was to look at the link between periodontal disease and the Healthy Eating Index (HEI), a method for assessing diet quality in accordance with the Dietary Guidelines for Americans recommendations with a higher score indicating better compliance. The study was performed using data from the National Health and Nutrition Examination Study, a nationally representative survey performed in 2-year cycles, from 2011 to 2012. The authors found that dietary structure was linked to the prevalence of periodontitis, and patients with a higher HEI were less likely to have periodontitis. However, the authors suggest future prospective longitudinal studies to confirm the causality observed.

A situation that poses potential risks to human health is the contamination of food by heavy metal(oids), as it can occur, for example, in the case of vegetables, particularly those grown in mining areas. However, the oral bioaccessibility and gingival cytotoxicity of heavy metals in wild plants are still not fully understood. Some of these plants are edible and comprise an important part of the diet in many countries. In this sense, Tian et al. evaluated the levels of total and bioaccessible Cr, As, Cd, Pb, and Ni in four wild vegetables in mining areas in southwest China. Furthermore, the authors studied the cytotoxicity and underlying mechanisms of plant extracts in human gingival epithelial cells. The results indicated that polluted vegetable intake caused toxic effects on human gingival cells, and the authors concluded that consuming wild vegetables from mining areas may cause adverse health effects on the human oral cavity.

According to WHO, “*Stunting is the impaired growth and development that children experience from poor nutrition, repeated infection, and inadequate psychosocial stimulation. Children are defined as stunted if their height-for-age is more than two standard deviations below the WHO Child Growth Standards median*” (3). Thus, failure to monitor growth in early childhood is potentially harmful to children on several levels. Verbal motor skills play an essential role in the oral function of the stomatognathic system, which includes mastication, swallowing and speech. Therefore, early attention to possible disorders of the oral function of the stomatognathic system can avoid complications in the nutritional status and quality of life of children. In their cross-sectional study, Sianturi et al. analyzed the correlation of oral function of the stomatognathic system in 58 stunting children, aged 7 to 12 years, with a history of growth deficit in childhood, and assessed the mastication, chewing, swallowing, and speech activities. The results showed that all children with short and very short stature showed unilateral mastication, dominant mastication without lip closure and slow chewing speed. In addition, malnutrition significantly affects all quality of life, including physical, emotional, social aspects, and the functioning of the stomatognathic system which is directly and indirectly related to the oral health of individuals.

Taken together, the studies included in this Research Topic are good contributions to the advancement of knowledge that relates nutrition and oral health and emphasize the importance of oral biology for health as a whole, beyond oral health.

## Author contributions

FCS prepared the main draft of this Editorial, which was reviewed by GB, EL, and PC. All authors contributed to the manuscript and approved the final and submitted version.
